# Unconventional approaches to mechanical ventilation—step-by-step through the COVID-19 crisis

**DOI:** 10.1186/s13054-020-02954-y

**Published:** 2020-05-18

**Authors:** Christopher Lotz, Quirin Notz, Peter Kranke, Markus Kredel, Patrick Meybohm

**Affiliations:** Department of Anesthesiology and Critical Care, University Hospital of Wuerzburg, University Wuerzburg, Oberduerrbacher Str. 6, 97080 Wuerzburg, Germany

Health care systems around the world face extreme challenges during the pandemic of SARS-CoV-2. It has been reported that up to 20% of the patients develop an acute respiratory distress syndrome (ARDS) and approximately 12% require mechanical ventilation. In many countries, this may lead to a rapid shortage of intensive care (ICU) ventilators. As such, a stepwise approach and triage utilizing all available types of ventilators might be necessary. This includes unconventional ideas that have been recently promoted in social media (https://www.youtube.com/watch?v=uClq978oohY, https://www.youtube.com/watch?v=eSVbwWANqRI). As uncertainties of the correct sequence of ventilator utilization seem to exist, we aim to provide a quick overview of the possibilities and shed some light on recently discussed ideas.

Under normal circumstances, all patients in the ICU requiring mechanical ventilation are ventilated with an intensive care ventilator. ICU ventilators provide the highest performance, fast responding efficient triggering mechanisms, and often a plethora of different ventilation modes to best suit the individual patient. However, anesthesia ventilators as the next step in line have made considerable technical progress. Their performance is comparable to ICU ventilators, in particular when using controlled ventilation modes. Current generation piston ventilators include fresh-gas decoupling to minimize volu- or barotrauma and offer pressure-support modes with sufficient triggering and pressurization even under low fresh-gas flows. As such, one should not hesitate to use them if ICU ventilators are not available. A current APSF/ASA Guidance on Purposing Anesthesia Machines as ICU Ventilators emphasizes this (https://www.asahq.org/in-the-spotlight/coronavirus-covid-19-information/purposing-anesthesia-machines-for-ventilators). Third in line are transport ventilators, which vary largely in performance according to generation and model. Many different models are marketed. The simplest pneumatic models are gas-driven pumps that provide 100% oxygen, control of rate and tidal volumes, and a pressure relief valve. On the other hand, new sophisticated transported ventilators offer a variety of modes including pressure-support ventilation and advanced monitoring. Turbine-driven transport ventilators even demonstrated performance comparable with that of ICU ventilators. However, as they are supplied by ambient air, they can only be used with 100% oxygen to prevent contamination of the device itself and its surroundings. This is a major downfall and limits their use to bridging, e.g., during the required testing of anesthesia ventilators. However, limited accuracy exists when prompted to deliver small tidal volumes (tidal volumes ≈ 50 ml). This would be required in small children [1, 2].

Unconventional, improvised, and desperate methods as recently emphasized on social media (https://www.youtube.com/watch?v=uClq978oohY, https://www.youtube.com/watch?v=eSVbwWANqRI) might be the next step if all of these resources are exhausted. The concept of supporting multiple patients with a single ventilator emerged in the aftermath of September 11, 2001. Neyman et al. created a setup where a single ventilator could deliver a sufficient tidal volume to four identical human lung simulators in parallel [3]. The concept was further supported by an animal experiment in which four sheep were successfully oxygenated for 12 h with a single ventilator [4]. There is also a case study reporting a one-ventilator technique during air medical transport of twin newborns [5] and an article that pressure controlled ventilation was simultaneously achieved in two healthy volunteers via mask ventilation [6]. However, Branson et al. further investigated this concept with detailed measurements of tidal volumes (V_T_) while varying the compliance and resistance. They found that four test lungs with different compliances (here 50–70 ml/cmH_2_O) received a wide fluctuation of V_T_ (257–621 ml) in parallel ventilation. Tidal volumes could not be controlled for each subject. The authors concluded that the concept of parallel ventilation for mass-casualty respiratory failure should not be supported [7]. This seems particularly true in case of a mass outbreak of SARS-CoV-2 and subsequent ARDS. Differences in lung compliance, required F_i_O_2_, and PEEP levels are paramount in these patients. Insufficient ventilation of one or more patients may be the consequence, which could go undetected as the monitored ventilation parameters reflect the whole group of patients.

It is of further importance to emphasize that in case of ICU ventilator shortage, the allocation of the ventilators to each patient requires triage. As clearly outlined by Emanuel et al., the allocation of resources cannot be done on a first come first served basis [8]. A triage committee might be the best answer to spread the burden of these difficult decisions [9]. However, exact knowledge of the individual cases is required. Ventilator triage would likely require switching of the ventilators during the course of treatment according to disease severity and stage as well as weaning capabilities, e.g., from anesthesia ventilator to ICU ventilator.

In conclusion, modern anesthesia ventilators as well as new-generation transport ventilators provide a valuable resource. In case of ICU ventilator shortage, this resource can and should be primarily used with a clear conscience in ARDS patients (Fig. 1). Furthermore, it must be emphasized that unconventional, improvised methods are only justified if all of these resources are exhausted as the risks go up and the quality of care rapidly declines.

**Fig. 1:**
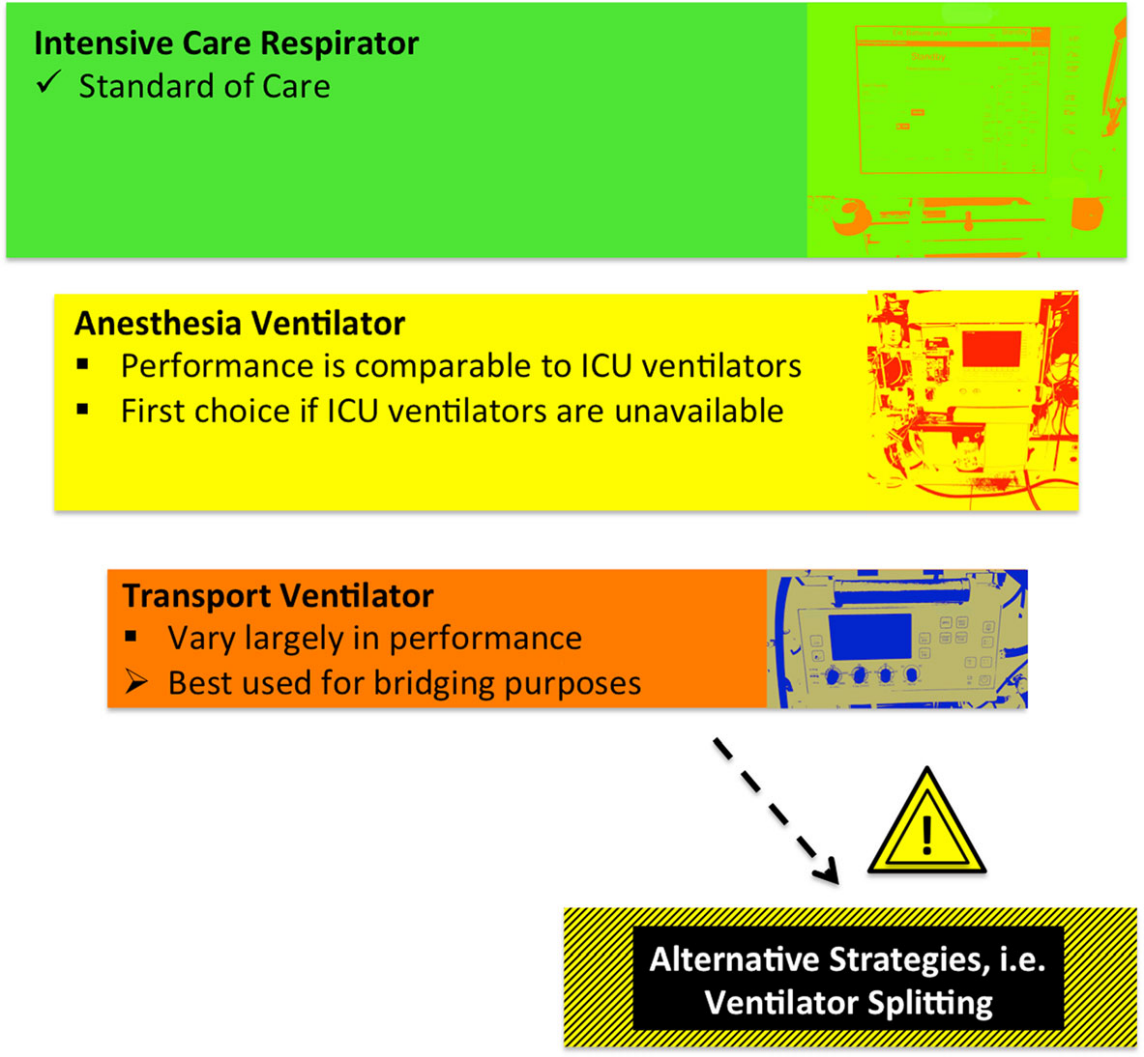
Although intensive care ventilators represent the standard of care, anesthesia ventilators can be used without difficulty if their conceptual differences are accounted for (e.g., the presence of trained personnel). Modern transport ventilators, albeit comparable in performance, can only be used for bridging as they are supplied by ambient air. Unconventional methods such as ventilator splitting should be treated with great caution and are only justified if all other resources are exhausted

## Data Availability

Not applicable.
